# Depression and Anxiety as Key Factors Associated With Quality of Life Among Lung Cancer Patients in Hai Phong, Vietnam

**DOI:** 10.3389/fpsyt.2019.00352

**Published:** 2019-05-16

**Authors:** Pham Minh Khue, Vu Thi Thom, Dao Quang Minh, Le Minh Quang, Nguyen Lam Hoa

**Affiliations:** ^1^Faculty of Public Health, Hai Phong University of Medicine and Pharmacy, Haiphong, Vietnam; ^2^Promotion 18, Lao Tropical Medicine and Public Health Institute, Vientiane, Laos; ^3^Department of General Surgery, Thanh Nhan Hospital, Hanoi, Vietnam; ^4^Oncology Center, Viet Tiep Hospital, Hai Phong, Vietnam; ^5^Department of Oncology, Hai Phong University of Medicine and Pharmacy, Haiphong, Vietnam

**Keywords:** tdepression, anxiety, quality of life, lung cancer, social support, Hai Phong, Vietnam

## Abstract

**Background:** Cancer is a leading cause of death. People living with cancer experience a variety of symptoms that might profoundly affect their quality of life (QoL).

**Objective:** The study aims to identify factors associated with the QoL of patients with lung cancer at the oncology department of Viet Tiep Hospital, Hai Phong city, Vietnam in 2018.

**Methods:** A cross-sectional study was conducted to collect data from lung cancer inpatients in Hai Phong city, Vietnam. The EQ-5D-5L and the EuroQol (EQ)-visual analogue scale (EQ VAS) were used to assess health-related quality of life (QoL). A multivariable regression analysis was performed on the EQ-5D utility score and the EQ VAS score as dependent variables, and socioeconomic, social support, and psychological factors as potential predictors.

**Results:** A total of 125 lung cancer patients were enrolled in this study. The highest proportion of respondents reporting any problems was in anxiety/depression (92.8%), pain/discomfort (81.2%), usual activities (75.2%), and mobility (60%) dimensions, while the lowest percentage was in self-care dimension (40.8%). The multivariate analyses showed that a low QoL score was significantly associated with depression, incapacity to pay, low response to treatment, and presence of side effects.

**Conclusion:** QoL of lung cancer patients is associated with anxiety/depression and other factors that can be modified by specific interventions. It is therefore possible to take care of psychological aspects to improve the QoL of Vietnamese people suffering from this condition.

## Introduction

Lung cancer is the leading cause of cancer death in men and the second leading death cause in women, with an estimated 1.6 million deaths in 2012 (1.1 million in men and 491,200 deaths in women) (1). In Vietnam, 21,667 deaths due to lung cancers representing 4.29% of total deaths were reported in 2017. The age adjusted death rate was estimated at 24.73 per 100,000 of the population, which placed Vietnam at the 37th position for lung cancer in the world (2). The survival rate of lung cancer patients remains poor with 1-year survival rate of 42% and 5-year survival rate of 16% (3). Lung cancer not only has a high mortality. It also has a high morbidity with a significant proportion of patients severely incapacitated by symptoms such as chest pain, cough, hemoptysis, and dyspnea (4). The assessment of patients with lung cancer ideally would therefore include two aspects: cancer outcomes and quality of life (QoL) outcomes. Cancer outcomes refer to the response to treatment, the duration of response and of symptom-free period, and the occurrence of relapse. QoL refers to the well-being of the patient once his or her diagnosis has been done (5–7).

Many factors might affect the QoL in cancer patients, including demographic factors (e.g., age, education, and household income) (8–10), social factors (e.g., support from family, friends, and others) (8, 10, 11), and psychological factors (such as depression and anxiety) (12–14). Specifically, based on a systematic review from 14 original studies of relationships between social support and QoL of lung cancer patients, it has been shown that social support received from family and friends affects positively the QoL particularly on its emotional and physical dimensions (11). Social support affects the QoL through two psychosocial mediating mechanisms: behavioral processes (e.g., fostering health-promoting behaviors, adherence) and psychological processes (e.g., stress appraisal) (15). Different components of the clinical process are also determinants of QoL, notably the time of cancer diagnosis, comorbidities, and the presence of side effects. Some studies have shown that the number of years since the cancer diagnosis was done is positively associated with the general QoL (16), and its role function (17) and social function dimensions (18). Poor QoL has been found to be associated with different conditions, particularly cardiovascular diseases and diabetes (19).

Among most relevant factors that affect patient’s QoL, psychological factors especially depression/anxiety have been shown to be constantly associated with the QoL. Cancer patients with depression have a poorer QoL (12–14, 20, 21). Anxiety has also been shown to be negatively associated with the different QoL dimensions (22). However, in Vietnam, QoL of patients with lung cancer has been poorly studied. Little is known about this outcome and its determinants. During the daily care of lung cancer patients, we found that patients usually suffer from depression and anxiety. In order to improve care to our patients, it is important to assess the association between these psychological factors and the patients’ QoL. From this perspective, we carried out this study among patients with lung cancer at the Department of Oncology of Viet Tiep Hospital, Hai Phong, Vietnam to identify factors associated with the QoL of patients.

## Methodology

### Study Design

A cross-sectional study was conducted to assess the QoL of lung cancer inpatients at the oncology department of Viet Tiep Hospital, Hai Phong city, Vietnam.

### Study Location

The study was conducted at Viet Tiep Hospital, one of the biggest hospitals in the northeast region of Vietnam. The Oncology Center of Viet Tiep Hospital is the only cancer treatment place in Hai Phong. Thus, almost all cancer patients have received treatment in there. According to the cancer registry, the annual cancer incidence is about 4,500–5,000 new patients since 2014 (23).

### Data Collection Period

Data collection was carried out between March and June 2018.

### Sample Size

We interviewed all patients diagnosed with lung cancer and being under treatment at the hospital during the data collection period.

### Eligibility Criteria

Inpatients were included in our study if they fulfilled the following inclusion criteria: being 18 years old or above, having a diagnosed lung cancer by biopsy and currently receiving cancer treatment in the hospital, being able to communicate, and willing to sign a written informed consent. Patients were excluded if they were unable to be interviewed, or if they refused to participate in the study.

### Data Collection Process

The researcher met with the inpatients diagnosed with lung cancer by biopsy and currently receiving treatment at the hospital. All participants were informed about the purpose of the research and the data collection process. The project was clearly explained. Patients could ask any questions regarding the project before signing the written informed consent form. Data were collected by face-to-face interview. Interviews took approximately 30 min.

### Instruments

Four questionnaires were used:

The EQ-5D-5L questionnaire was used to measure the QoL. The questionnaire consists of two parts: the EQ-5D-5L descriptive system and the EQ Visual Analogue scale (EQ VAS). The descriptive system contains five dimensions (mobility, self-care, usual activities, pain/discomfort, anxiety/depression). In the current study, due to the unavailability of validated Vietnamese scores, we used the Thailand scores to calculate the utility score of the study participants (24). The EQ VAS records the respondent’s self-rated health on a visual analogue scale with endpoints labeled “the best health you can imagine” and “the worst health you can imagine.” The Vietnamese version of EQ-5D-5L was validated in Vietnam (25, 26).

Social support status was measured with the Perceived Social Support Scale (PSSS). The scale consists of 12 items to measure the perception of support by the family, friends, and others. The rating ranges from 1 (totally disagree) to 7 (totally agree). The mean of scales is then calculated. A mean score <2.9 was considered as low support; a score of 3 to 5 was considered as moderate support; a score from 5.1 to 7 was considered as high support (27). The questionnaire was validated in Vietnamese. Cronbach’s alpha was found to be between 0.95 and 0.97 for the four subscales and 0.97 for the overall scale (28).

The Hospital Anxiety and Depression Scale (29) contains 14 items. It provides a brief measure of both anxiety (seven items) and depression (seven items) as two distinct subscales. Questions refer to the patient’s feelings during the past week. The seven items in each of the anxiety and depression subscales are scored from 0 to 3, resulting in subscale scores that range from 0 to 21. Two cut-off points were suggested by the original authors: 8 is the threshold of a possible case, and 10 is the threshold of a significant distress related to anxiety or depression (29). The questionnaire has been validated in Vietnam. It showed a Cronbach’s alpha of 0.79 for Hospital Anxiety and Depression Scale-Anxiety subscale (HADS-A) and 0.7 for Hospital Anxiety and Depression Scale-Depression subscale (HADS-D) (30).

A last questionnaire was used to collect basic sociodemographic information including gender, age, living milieu, capacity to pay treatments, etc.

### Statistical Analyses

Data were entered in Epidata version 1, and analyses were done with STATA version 12. Descriptive statistics were used to summarize the distribution of demographic, clinical data, social support, psychological factors related to depression/anxiety, EQ-5D-5L profiles, utility score, and visual analogue scale (VAS) scores. These included frequencies, mean, median, range, and standard deviations (SDs). Total scale score and subscale scores on measures were summed. The EQ-5D utility score was calculated using the EQ-5D-5L Crosswalk Index Value Calculator.

Utility and VAS scores had a normal distribution. The comparison between independent groups was therefore assessed with a *t*-test (for gender, economic, health insurance, placement, chronic disease situation, life threatening, effectiveness of treatment, and side effect) and ANOVA tests (for age groups, marital status, education level, social support, and psychological factors).

Bivariate analyses were performed between potential independent variables and the dependent variables. Variables whose association were significant at a value of *p* < 0.2 were retained for the multivariate analyses. Multivariate linear regression analyses were performed to quantify the unique contribution of each potential predictor on utility. The considered level of statistical significance was a *p*-value of 0.05 or less. Data used to support the findings of this study are available from the **Supplementary Material**.

## Results

A total of 125 participants with lung cancer were enrolled in the study. Most of the participants were male (72.0%) and 55 years old or above (82.40%). Half of the respondents (52.80%) had achieved a secondary education level. Half (57.60%) were unemployed. More than half of the participants said they did not have enough money for their needs in life (60.80%). Approximately 50% of the respondents thought that their disease had improved since the beginning of the treatment. More than half of the participants consider having a high social support (62.40%; **Table 1**).

**Table 1:** Demographic and clinical characteristics of participants (*n* = 125).

Variables	*n* (%)
**Age in years **	
Mean (SD)	61.07 (9.09)
35–44	7 (5.60)
45–54	15 (12.0)
55–64	53 (42.40)
>64	50 (40.0)
**Sex**	
Male	90 (72.0)
Female	35 (28.0)
**Profession**	
Unemployed	75 (80.0)
Employed	25 (20.0)
**Number of children**	
Mean (SD)	2.75 (1.25)
**Education**	
Primary	21 (16.80)
Secondary	66 (52.80)
College and more	36 (30.40)
**Capacity to pay treatments**	
Not enough	76 (60.80)
Enough	49 (39.20)
**Health insurance status**	
No	4 (3.20)
Yes	121 (87.20)
**Live with others**	
No	5 (4.0)
Yes	120 (96.0)
**Place**	
Countryside	97 (77.60)
City	28 (22.40)
**Months of cancer diagnosis**	
Mean (SD)	15.71 (20.43)
**Chronic disease**	
No	99 (79.20)
Yes	26 (20.80)
**Life threatening in the near future**	
No	8 (6.40)
Yes	117 (93.60)
**Response to treatment**	
Improved	27 (21.60)
Improved a little	38 (30.40)
No change	35 (28.0)
Deteriorated	25 (20.0)
**Feeling about treatment side effect**	
Unbearable	18 (14.40)
Bearable	107 (85.60)
**Frequency of social support**	
Low support	4 (3.20)
Moderate support	43 (34.40)
High support	78 (62.40)

**Table 2** illustrates that although 26.40% of participants are likely to have a depression, more than half of the participants should be evaluated for this mental disease. Two-thirds of the participants should be evaluated for an anxiety disorder.

**Table 2:** Prevalence of a suspicion of depression and anxiety (*n* = 125).

Scale score	Depression		Anxiety	
	*N*	%	*N*	%
Likely noncase	47	37.60	43	34.40
Doubtful case	45	36.0	43	34.40
Likely case	33	26.40	39	31.20

**Table 3** depicts the profiles of EQ-5D-5L domains according to the frequencies of each item’s response. The highest proportion of respondents reporting any problems was in the anxiety/depression dimension (92.8%), followed by pain/discomfort (81.2%), the usual activities (75.2%), and mobility (60%) dimensions, while the lowest percentage was found in the self-care dimension (40.8%).

**Table 3:** EQ-5D-5L: frequencies by dimension and level, as well as by sex (*n* = 125).

Variables	Total *n *(%)	Age groups				Sex	
		35–44 *n* (%)	45–54 *n* (%)	55–64 *n* (%)	65+ *n* (%)	Male *n* (%)	Female *n* (%)
**Mobility**							
Level 1	50 (40.0)	4 (57.40)	10 (66.67)	20 (37.74)	16 (32.0)	41 (45.56)	9 (25.71)
Level 2	38 (30.40)	3 (42.86)	3 (20.0)	17 (32.08)	15 (30.0)	25 (27.78)	13 (37.14)
Level 3	24 (19.20)	0 (0.0)	2 (13.33)	7 (13.21)	15 (30.0)	17 (18.89)	7 (20.0)
Level 4	9 (7.20)	0 (0.0)	0 (0.0)	5 (9.43)	4 (8.0)	4 (4.44)	5 (14.29)
Level 5	4 (3.20)	0 (0.0)	0 (0.0)	4 (7.54)	0 (0.0)	3 (3.33)	1 (2.86)
**Self-care**							
Level 1	74 (59.2)	7 (100.0)	12 (80.0)	30 (56.6)	25 (50.0)	56 (62.22)	18 (51.43)
Level 2	21 (16.8)	0 (0.0)	1 (6.67)	9 (19.98)	11 (22.0)	16 (17.78)	5 (14.29)
Level 3	20 (16.0)	0 (0.0)	2 (13.30)	7 (13.21)	11 (22.0)	12 (13.33)	8 (22.86)
Level 4	5 (4.0)	0 (0.0)	0 (0.0)	3 (5.66)	2 (4.0)	3 (3.33)	2 (5.71)
Level 5	5 (4.0)	0 (0.0)	0 (0.0)	4 (7.55)	1 (2.0)	3 (3.33)	2 (5.71)
**Usual activity**							
Level 1	31 (24.8)	2 (28.57)	8 (53.3)	11 (20.75)	10 (20.0)	23 (25.56)	8 (22.86)
Level 2	46 (36.8)	4 (57.14)	5 (33.33)	19 (35.85)	18 (36.0)	34 (37.78)	12 (34.29)
Level 3	27 (21.6)	1 (14.29)	2 (13.34)	12 (22.64)	12 (24.0)	21 (23.33)	6 (17.14)
Level 4	14 (11.2)	0 (0.0)	0 (0.0)	6 (11.32)	8 (16.0)	8 (8.89)	6 (17.14)
Level 5	7 (5.6)	0 (0.0)	0 (0.0)	5 (9.44)	2 (4.0)	4 (4.44)	3 (8.57)
**Pain/discomfort**							
Level 1	23 (18.80)	1 (14.29)	2 (13.33)	12 (22.64)	8 (16.0)	21 (23.33)	2 (5.71)
Level 2	60 (48.0)	5 (71.42)	10 (66.67)	21 (39.63)	24 (64.0)	40 (44.44)	20 (57.14)
Level 3	30 (24.0)	1 (14.29)	2 (13.33)	12 (22.64)	15 (30.0)	22 (24.44)	8 (22.86)
Level 4	10 (8.0)	0 (0.0)	1 (6.67)	6 (11.32)	3 (6.0)	5 (5.56)	5 (14.29)
Level 5	2 (1.60)	0 (0.0)	0 (0.0)	2 (3.77)	0 (0.0)	2 (2.22)	0 (0.0)
**Anxiety/depression**							
Level 1	9 (7.2)	0 (0.0)	1 (6.67)	3 (5.66)	5 (8.93)	9 (10.0)	0 (0.0)
Level 2	67 (53.6)	5 (71.43)	11 (73.33)	25 (47.17)	26 (46.63)	48 (53.33)	19 (54.29)
Level 3	39 (31.2)	2 (28.57)	2 (13.33)	20 (37.74)	20 (35.71)	29 (32.22)	10 (28.57)
Level 4	10 (8.0)	0 (0.0)	1 (6.67)	5 (9.43)	5 (8.93)	4 (4.44)	6 (17.14)

**Table 4** displays the results of the univariate analyses on the potential determinants of utility in the participants. Overall, our analysis showed that the mean scores for EQ-5D utility and EQ VAS were statistically significantly different for the various categories of the following variables: capacity to pay (*p* < 0.001 and *p* = 0.0001, respectively), perception of the disease being life threatening (*p* < 0.0031 and *p* = 0.014, respectively), response to treatment (*p* < 0.0001 and *p* < 0.0001, respectively), presence of side effects (*p* < 0.0001 and *p* = 0.0015, respectively), having social support (*p* = 0.0001 and *p* < 0.0001, respectively), having a likely depression (*p* < 0.0001 and *p* < 0.0001, respectively), and having a likely anxiety (*p* < 0.0001 and *p* < 0.0001, respectively).

**Table 4:** Results of univariate analyses of utility scores.

Variables	EQ-5D utility				EQ VAS			
	Mean	SD	F/*t*	*p*-value	Mean	SD	F/*t*	*p*-value
**All patients (*n* = 125)**	0.53	0.26			48.5	20.98		
**Sociodemography**								
**Capacity to pay**								
Not affordable	0.46	0.24			42.89	18.29		
Affordable	0.65	0.25	−4.26	<0.0001**	57.18	22.09	−3.93	0.0001**
**Life threatening**								
No	0.79	0.14	3.02		65.88	19.65		
Yes	0.51	0.26		0.0031**	47.31	20.62	2.47	0.0148**
**Response to treatment**								
No change/deterioration	0.42	0.28			39.45	18.64		
Improvement	0.63	0.20	−4.72	<0.0001**	56.85	19.63	−5.07	<0.0001**
**Side effects**								
Unbearable	0.27	0.3			34.17	19.50		
Bearable	0.57	0.23	−4.97	<0.0001**	50.91	20.33	−3.25	0.0015**
**Social support**								
Low support	0.45	0.15			48.75	13.15		
Moderate support	0.40	0.32			35.23	17.18		
High support	0.60	0.19	10.67	0.0001*	55.79	19.72	16.68	<0.0001*
**Psychology factors**								
**Depression**								
Likely noncase	0.70	0.15			65.74	14.76		
Doubtful case	0.52	0.24			45.22	16.41		
Likely case	0.31	0.24	31.16	<0.0001*	28.39	12.39	63.30	<0.0001*
**Anxiety**								
Likely noncase	0.72	0.15			67.09	15.77		
Doubtful case	0.52	0.20			43.84	15.92		
Likely case	0.33	0.27	31.4	<0.0001*	33.13	14.94	51.60	<0.0001*

EQ VAS, EQ-visual analog scale; EQ-5D, EuroQol-5 Dimensions.

*ANOVA test; **t-test.

The association between potential predictors of EQ-5D utility scores and EQ VAS scores were then analyzed by using a multiple linear regression. **Table 5** shows that the final model retains four predictors (capacity to pay, response to treatment, side effects, and depression) of utility score while two predictors (response to treatment, depression) are retained for the EQ VAS scores.

**Table 5:** Multiple linear regression on EQ-5D utility score and EQ VAS scores.

Variables	EQ-5D utility score*			EQ VAS**		
	Coef.	95% CI	*p*-value	Coef.	95% CI	*p*-value
**Capacity to pay **						
Not affordable	1.00					
Affordable	0.09	0.01; 0.16	0.027	NS		
**Response to treatment**						
No change/deterioration	1.00			1.00		
Improvement	0.11	0.03; 0.18	0.004	9.27	3.96–14.58	0.001
**Side effects**						
Unbearable	1.00					
Bearable + not important	0.16	0.05; 0.27	0.012	NS		
**Depression**						
Likely noncase	1.00			1.00		
Doubtful case	−0.10	−0.19; −0.01	0.023	−17.73	−23.81; −11.66	<0.001
Likely case	−0.26	−0.35; −0.15	<0.001	−23.88	−40.68; −27.35	<0.001

*(Prob> F = 0.0000, R-squared = 0.45, Adj R-squared = 0.43).

**(Prob> F = 0.0000, R-squared = 0.55, Adj R-squared = 0.54).

NS, not significant.

## Discussion

To our knowledge, our study is the first study on the QoL of Vietnamese patients with lung cancer. Our results show that the most frequently EQ-5D identified problems were anxiety/depression and pain/discomfort. These findings are similar to what has been found in a previous study conducted in Indonesia with the same instrument, but another type of cancer (cervical cancer) (31). One notes that a previous study in Vietnam with the same instrument, but another disease, HIV/AIDS, also found that the most affected QoL dimension was anxiety/depression (27.5%). These two studies underline the importance, when dealing with a chronic disease that affects physical health, of considering the mental health of the patient. Treating a physical disease should not be limited to the use of treating the etiology of the condition. It should also include the search for mental impacts of the disease. An effective management of a chronic disease requires the collaboration of physicians specialized in the disease with psychiatrists or doctors familiar with mental diseases.

The literature reveals that utility scores in lung cancer patients vary across countries. Our study found a mean (SD) utility score of 0.53 and an EQ VAS score of 48.5. A study performed in Italy found values of 0.58 (32). In France and Germany, values were 0.58 and 58.0, respectively (33). In the United States, United Kingdom, and Canada, the scores were 0.67, 0.76, and 68, respectively (34, 35). The differences of utility scores might reflect differences in weight put on the different dimensions among countries. They might also reflect differences in treatment protocol that lead to different degrees of impairment (36–38). Yet, we have to remember that our study didn’t use utility values validated for the Vietnamese population. We cannot reject the possibility that the use of validated values would have changed our results (39, 40). Formally, it is desirable that further studies on QoL in Vietnam are preceded by validation studies of the utility instrument that might be used.

Another important observation from our study is that one-third of respondents with lung cancer might suffer from depression/anxiety. Of course, this suspicion should be validated with the diagnostic questionnaires used in psychiatry. Nevertheless, the high prevalence of possible depression/anxiety in cancer patients is alarming. It could be explained by the side effects of the treatment process. But other explanations should also be explored. For example, the cost of cancer treatment is expensive. Thus, patients suffer from economic pressure. This can lead to anxiety and depression. Additionally, the capacity of working is decreased in people with cancer, adding to the economic burden that patients have to face. Another factor could be the perception that the disease will lead to death in the near future. Depression and anxiety are multifactorial outcomes. This is suggested by studies that show that even in countries where physical and mental problems are usually jointly addressed, such as the Czech Republic or Japan, anxiety and depression can be found in a high proportion of patients compared to our study, in nearly half of the people with lung cancer (14, 22). This underlines the importance not only to treat medically these mental conditions but also to look for the factors that might explain anxiety and depression. The importance of depression is still apparent in the multivariate analyses. In summary, our results highlight what has been said above: management of anxiety and depression in lung cancer patients should be part of the general management of lung cancer in Vietnam.

Our study has several limitations. First, it was based on a cross-sectional design. Therefore, although our predictors are likely to be real predictors, no causal conclusion can formally be drawn. Secondly, our sample was limited to 125 patients who were currently receiving the treatment at Viet Tiep Hospital. This number of patients participating into the survey is rather small, and we cannot assure that the sample is representative of Vietnamese cancer patients. The generalizability of our finding is therefore limited. We have involved at the time of our research all the patients who are under treatment in Viet Tiep Hospital, the biggest hospital of northeast area of Vietnam. Finally, a major limitation of our study is unavailability of validated EQ-5D values of the Vietnamese population. Using Thai value might not be very adequate. In spite of these limitations, our research found the impact of treatment results, side effects, the patients’ capacity to pay, and the patients’ mental health problems such as depression/anxiety on the QoL of Vietnamese lung cancer patients. The most important result is that depression/anxiety can affect the patient’s QoL, and this is an often neglected condition that can, nevertheless, be treated effectively and at very low costs in Vietnam.

## Recommendations

Based on our results, we can propose that every lung cancer patient in Vietnam should be evaluated for the presence of depression and anxiety and be offered treatment for these conditions, if they occur as comorbidity.

## Ethics Statement

The study protocol approval was obtained from the Institution Review Board of Haiphong University of Medicine and Pharmacy, and data collection process was approved by the Oncology Center of Viet Tiep Hospital. The objectives of the study were clearly explained to cancer patients, and informed consent was obtained. All information is confidential. All data were collected anonymously.

## Author Contributions

PK designed the study, supervised the data collection, led the data analysis, and drafted the manuscript. VT participated in the study design, questionnaire construction, interpretation of the results, and manuscript writing. DM participated in the coordination of the data collection, data analysis, data interpretation, and in revising the manuscript. LQ participated in the study design, interpretation of the results, and manuscript writing. NH guided the design of the study, submitted the protocol for ethical review, and chaired the manuscript writing. All authors read and approved the final manuscript.

## Funding

This work originated from a student master thesis work supported by Lao Tropical and Public Health Institute, Vientiane, Laos and Haiphong University of Medicine and Pharmacy, Vietnam.

## Conflict of Interest Statement

The authors declare that the research was conducted in the absence of any commercial or financial relationships that could be construed as a potential conflict of interest.
